# A Heart Failure-Associated *SCN5A* Splice Variant Leads to a Reduction in Sodium Current Through Coupled-Gating With the Wild-Type Channel

**DOI:** 10.3389/fphys.2021.661429

**Published:** 2021-03-22

**Authors:** Yang Zheng, Xiaoping Wan, Dandan Yang, Angelina Ramirez-Navarro, Haiyan Liu, Ji-Dong Fu, Isabelle Deschênes

**Affiliations:** ^1^Department of Physiology and Cell Biology, Frick Center for Heart Failure and Arrhythmias, Davis Heart and Lung Research Institute, The Ohio State University, Columbus, OH, United States; ^2^Department of Biomedical Engineering, Case Western Reserve University, Cleveland, OH, United States

**Keywords:** cardiac sodium channel, heart failure, arrhythima, electrophyiology, patch – clamp technique

## Abstract

Na_v_1.5, encoded by the gene *SCN5A*, is the predominant voltage-gated sodium channel expressed in the heart. It initiates the cardiac action potential and thus is crucial for normal heart rhythm and function. Dysfunctions in Na_v_1.5 have been involved in multiple congenital or acquired cardiac pathological conditions such as Brugada syndrome (BrS), Long QT Syndrome Type 3, and heart failure (HF), all of which can lead to sudden cardiac death (SCD) – one of the leading causes of death worldwide. Our lab has previously reported that Na_v_1.5 forms dimer channels with coupled gating. We also found that Na_v_1.5 BrS mutants can exert a dominant-negative (DN) effect and impair the function of wildtype (WT) channels through coupled-gating with the WT. It was previously reported that reduction in cardiac sodium currents (I_Na_), observed in HF, could be due to the increased expression of an *SCN5A* splice variant – E28D, which results in a truncated sodium channel (Na_v_1.5-G1642X). In this study, we hypothesized that this *SCN5A* splice variant leads to I_Na_ reduction in HF through biophysical coupling with the WT. We showed that Na_v_1.5-G1642X is a non-functional channel but can interact with the WT, resulting in a DN effect on the WT channel. We found that both WT and the truncated channel Na_v_1.5-G1642X traffic at the cell surface, suggesting biophysical coupling. Indeed, we found that the DN effect can be abolished by difopein, an inhibitor of the biophysical coupling. Interestingly, the sodium channel polymorphism H558R, which has beneficial effect in HF patients, could also block the DN effect. In summary, the HF-associated splice variant Na_v_1.5-G1642X suppresses sodium currents in heart failure patients through a mechanism involving coupled-gating with the wildtype sodium channel.

## Introduction

The voltage-gated cardiac sodium channel, known as Na_v_1.5 and encoded by the *SCN5A* gene, is the predominant voltage-gated sodium channel expressed in the heart. The channel initiates the cardiac action potential in myocytes by generating a rapid sodium influx. Dysfunction in Na_v_1.5 can lead to various cardiac arrhythmic diseases. Mutations in *SCN5A* have been linked to several inherited cardiac channelopathies including Long QT Syndrome Type 3 and Brugada syndrome (BrS) (Amin et al., 2010; Veerman et al., 2015). In addition to congenital disorders, reduction in sodium current, I_Na_, was linked to human heart failure (HF) (Tomaselli and Zipes, 2004). However, the mechanisms leading to a reduction in I_Na_ are not fully understood. Pathological behaviors of the channel such as (1) reduction in sodium channel protein due to degradation or decrease in mRNA, (2) lower surface expression level and (3) abnormal kinetics such as changes in voltage-dependence of channel gating can all contribute to the decreased I_Na_. Our lab previously demonstrated that cardiac sodium channels exist and gate as dimers and importantly, we showed that some Na_v_1.5 mutants exert dominant negative (DN) effect on the wildtype channel (WT) leading to a reduction in current through coupled-gating with the WT (Clatot et al., 2017). Notably, we were able to uncouple the channels by inhibiting the 14-3-3 accessory protein, resulting in removal of the DN effect (Clatot et al., 2017, 2018). We and the Lampert lab also showed a similar mechanism for the neuronal voltage-gated sodium channels Na_v_1.1, Na_v_1.2 and Na_v_1.7 (Clatot et al., 2017; Rühlmann et al., 2020). Therefore, uncoupling the Na_v_1.5 or other Na_v_s from their pathogenic mutants could serve as a new strategy to develop clinical treatments for cardiac or neuronal diseases associated with Na_v_s dysfunction.

There are also cardiac pathologies, such as HF, where decreases in I_Na_ are observed but without being linked to *SCN5A* mutations. It is very likely that the phenotypes can be associated with post-transcriptional and post-translational modifications of *SCN5A*. Alternative splicing or differential splicing is a post-transcriptional modulation of a gene that generates different combination of the translated regions, yielding different splice variants of the expressed gene. *SCN5A* splice variants have been studied for their implication in cardiac pathogenesis, and the C-terminal splice variants were recently shown to play a role in human cardiac pathology. Three C-terminal splice variants, E28B, E28C and E28D are associated with human HF, and were shown to activate unfolded protein response (Shang et al., 2007, 2008; Gao et al., 2013; Noyes et al., 2017). Among them, E28D is the most abundant one which gets significantly upregulated in human HF, and is also related with pulmonary arterial hypertension in clinical practice (Banerjee et al., 2020). *SCN5A*-E28D generates a non-functional truncated sodium channel Na_v_1.5-G1642X. This splice variant not only results in a nonfunctional channel but was also shown to reduce the WT current. In this study, we therefore aimed to investigate the dominant negative effect mechanisms produced by the E28D splice variant. Based on our earlier findings demonstrating dimerization and biophysical coupling of sodium channels, we hypothesized that this *SCN5A* splice variant leads to a reduction in I_Na_ through biophysical coupling with the WT. Our team previously found that the common Na_v_1.5-H558R can interact with multiple pathogenic *SCN5A* genetic mutations found in BrS or Long QT syndrome to rescue their pathogenic effect (Ye et al., 2003; Shinlapawittayatorn et al.,2011a,b). Interestingly, this Na_v_1.5-H558R polymorphism was reported to improve the survival of HF patients (Aleong et al., 2005). We therefore also explored if the presence of this polymorphism could influence the DN-effect produced by the splice variant.

## Materials and Methods

### Na_v_1.5 Constructs

The *SCN5A* splice variant G1642X, polymorphism H558R, and mutation S460A plasmids were created using the QuickChange II XL Site-Directed Mutagenesis Kit (Agilent Technologies) according to the manufacturer’s instruction. The constructs were made on the pcDNA3.1 vector containing the N-terminus GFP-fused Na_v_1.5 (Clatot et al., 2012) (BD Biosciences, San Jose, CA, United States). The outcomes were verified by sequencing.

### Cell Culture and Transfection

The plasmids of interest including the cardiac sodium channels and the 14-3-3 inhibitor difopein were transiently transfected into HEK 293 cells and human induced pluripotent stem cells derived cardiomyocytes (hiPSC-CMs). HEK 293 cells were maintained in DMEM supplemented with 10% FBS and 1% penicillin-streptomycin until they grew to about 60% confluence and were then used for transfection. During the transfection, the cells were maintained in DMEM without penicillin-streptomycin. The transfection was done with the reagent FUGENE^®^ (Promega, Madison, WI, United States) according to the manufacturer’s instruction. The total amount of plasmid transfected into HEK293 cells was 0.6 μg for patch-clamp experiments; however, for the experiments where difopein was used, an additional 0.6 μg of difopein plasmids was transfected or 0.6 μg of empty pcDNA3.1 vector for the control group in order to balance the total amount of transfected DNA. For the biochemical experiments a total of 2.0 μg of the plasmids of interest were transfected into HEK293 cells. In the group where two plasmids of interest were co-expressed, i.e., WT and G1642X, 0.3 μg (for patch-clamp) or 1.0 μg (for biochemistry) of each DNA was transfected. In the group where only one sodium channel was expressed, 0.3 μg (for patch-clamp) or 1.0 μg (for biochemistry) of empty pcDNA3.1 vector was transfected in order to balance the total amount of transfected DNA. For hiPSC-CMs experiments, we used the commercially available iCells^®^ (FujiFilm, Madison, WI, United States). The iCells^®^ were cultured according to manufacturer’s instruction. We transfected 1.8 μg of Na_v_1.5-G1642X for the experimental group and 1.8 μg of YFP for the control group. The iCells^®^ were transfected using Lipofectamine 2000 (Invitrogen, Carlsbad, CA, United States) according to the manufacturer’s instruction.

### Electrophysiology Measurement

Sodium currents were recorded at room temperature (21–23°C) by patch-clamp technique in whole-cell configuration, 24 h (HEK 293 cells) and 48 h (iCells^®^) after the transfection. The recordings were obtained using the Axopatch 200A amplifier (Molecular Devices, San Jose, CA, United States) and the Digidata 1440A digitizer (Molecular Devices, San Jose, CA, United States). The protocols were generated with the pCLAMP 14.2 software (Molecular Devices, San Jose, CA, United States). For HEK 293 cells the intracellular solution contained: NaCl 35 mM, CsF 105 mM, EGTA 10 mM, and Cs-HEPES 10 mM and the extracellular solution contained: NaCl 135 mM, KCl 4.5 mM, MgCl_2_ 0.7 mM, CaCl2 2 mM, glucose 10 mM, and HEPES 10 mM. For the iPSC-CMs recording the intracellular solution contained: CsMes 130mM, TEACl 20 mM, MgCl_2_ 1 mM, EGTA 10 mM, HEPES 10 mM, and MgATP 4 mM. The extracellular solution contained: NaCl 25 mM, CsCl 5.4 mM, MgCl_2_ 1.8 mM, CaCl_2_-2H_2_O 1.8 mM, HEPES 10 mM, Glucose 10 mM and NMDG 105 mM. The pH of the iPSC-CM intracellular solution is adjusted to 7.2, and for the other solutions the pH was adjusted to 7.4. The current-voltage relationships of the sodium currents were recorded by holding the resting membrane potential at −120 mV and stepping from −80 mV to +60 mV in 10 mV interval (each step hold for 30 ms). The persistent or late sodium currents (I_Na–late_) were recorded with a test pulse of 250 ms at −30 mV from a holding potential of −120 mV. Steady-state inactivation and recovery from inactivation were recorded using protocols as previously described (Poelzing et al., 2006). Briefly, the recovery from inactivation was recorded with a two-pulse protocol. Both the pre-pulse and the test-pulse duration are 30ms, stepping from −30 mV to −120 mV. The interval between the two pulses ranges from 1.8 ms to 70 ms. Currents of the recovery from inactivation were fit to the following equation:

$$I_{t\,e\,s\,t}/I_{p\,r\,e-p\,u\,l\,s\,e}=1-{\text{e}}^{-t/\tau_{r\,e\,c}}$$

The steady-state inactivation was studied with a 500 ms pre-pulse ranging from −140 mV to −30 mV, followed by a 30ms test pulse stepping from −120 mV to −30 mV. The currents for the steady-state inactivation were fit to a Boltzmann distribution using the following equation:

$$I/I_{m\,a\,x}={\left(1\,e^{\left(V-V_{1/2}\right)/k_{v}}\right)}^{-1}$$

The fitting curves of steady-state inactivation and recovery from inactivation analysis were generated with Origin 10.1.1 software (OriginLab Corporation, Northampton, MA, United States). Throughout the whole-cell patch-clamp recordings, about 85% of the series resistance was compensated with a lag of about 10 μs. Leak subtraction protocol was used only for the persistent sodium current recordings.

### Biochemical Analysis

HEK 293 cells were transfected with a total amount of 2.0 μg of DNA for the biochemical analysis. The cells were washed 48 h after transfection and collected for the surface biotinylation assays or co-immunoprecipitation (co-IP) experiments. Cell surface biotinylation assays were performed according to the manufacturer’s instruction and as previously described (Hoshi et al., 2014; Clatot et al., 2018). Briefly, the surface proteins were labeled at 4°C for 30 min with 0.25mg/ml Pierce Sulfo-NHS-SS-Biotin (Thermo Fisher Scientific). The biotinylated proteins were then isolated through a Pierce NeutrAvidin Agarose resin column (Thermo Fisher Scientific). Co-IP experiments were performed with Dynabeads Protein G (Thermo Fisher Scientific) as previously described (Clatot et al., 2012, 2017). Briefly, the magnetic beads were washed twice with citrate-phosphate buffer (pH 5.4) and then incubated with 5 μg rabbit anti-GFP antibody (Invitrogen, Cat# A11122) for 2 h at room temperature. The incubated beads were then washed with citrate-phosphate buffer plus 0.1% Tween20 and incubated with precleared lysate sample (total amount of 400 mg protein) at 4°C overnight. Next, the sample was washed three times with lysis buffer before elution. The proteins were eluted with XT sample buffer (Bio-Rad) at 37°C for 1 h. Finally, the western blot experiments were performed to reveal the results of both biotinylation assays and co-IPs using the following primary antibodies: anti-Na_v_1.5 antibody 1:1000 (kind gift from Dr. Thomas Hund) (Hund et al., 2010), anti-HA antibody 1:1000 (Sigma, Cat# H6908), and anti-GFP antibody 1:2000 (Invitrogen, Cat# A11122).

### Statistical Analysis

Statistical analyses were performed using the standard statistical package in Origin 10.1.1 (OriginLab Corporation, Northampton, MA, United States). Student’s t-test was performed at a significance level *p* = 0.05 for single comparison after a normality test with the Shapiro-Wilk method for sample size 7–50. Two-side p values less than 0.05 were considered statistically significant. Results were presented as mean ± SEM.

## Results

### Na_v_1.5-G1642X Exert DN Effect When Co-expressed With the WT

**FIGURE 1:**
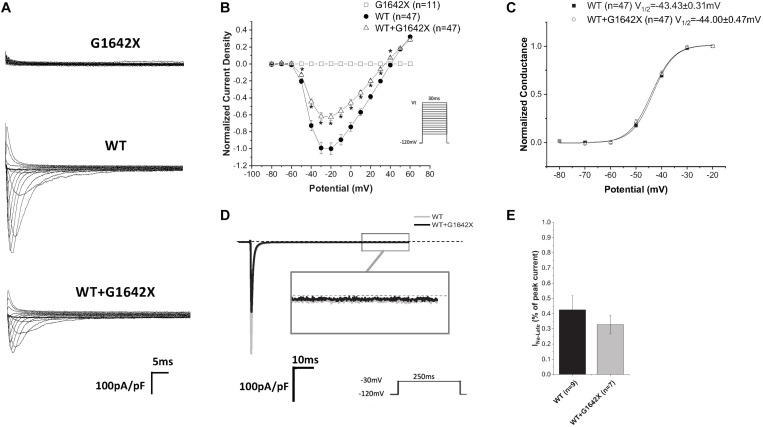
**(A)** Representative sodium current traces elicited using the indicated voltage protocol. **(B)** Current voltage relationships (I/V) curves. **(C)** Voltage-dependent conductance curves for Na_v_1.5-WT, Na_v_1.5-G1642X, and the co-expression of Na_v_1.5-WT and Na_v_1.5-G1642X recorded from HEK 293 cells. **(D)** Representative late sodium currents. **(E)** Quantification of the late sodium currents recorded from Na_v_1.5-WT and the co-expression of Na_v_1.5-WT with Na_v_1.5-G1642X. Voltage protocols used are included. *p < 0.05 compared to Na_v_1.5-WT.

**FIGURE 2:**
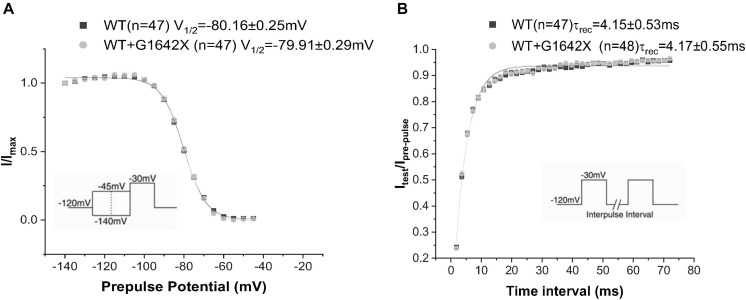
Recovery from inactivation **(A)** and steady-state inactivation **(B)** recorded from HEK 293 cells expressing Na_v_1.5-WT or Na_v_1.5-WT+Na_v_1.5-G1642X. The splice variant G1642X does not influence the recovery from inactivation and the steady-state inactivation of the Na_v_1.5-WT. Voltage protocols used are included.

The *SCN5A*-E28D splice variant results in a Na_v_1.5 truncated at the 1642th glycine residue therefore truncating the channel from the middle of the DIV S4 segment until the end of the C-terminus (Shang et al., 2007). It was previously shown that co-expression of this truncated splice variant with the WT channel leads to a reduction in WT currents (Shang et al., 2007). In order to study the mechanism by which Na_v_1.5-G1642X exerts this DN effect, we recorded sodium currents using whole-cell patch-clamping. We first confirmed that the splice variant is non-functional (*n* = 11) (Figures 1A,B). When Na_v_1.5-G1642X was co-expressed with the WT, the WT current was significantly (*n* = 47, *p* < 0.05) reduced by Na_v_1.5-G1642X although we transfected the same amount of WT DNA into each group, therefore suggesting a DN-effect (Figures 1A,B). For a DN effect, a reduction of 75% in current density would be expected when co-expressing the same level of WT and splice variant (Clatot et al., 2017), however, here the current was reduced by about 40% (Figures 1A,B). This could be explained by a partial degradation of the truncated Na_v_1.5-G1642X. We then quantified the expression level of the Na_v_1.5-WT and Na_v_1.5-G1642X, and found that about 40% of the truncated Na_v_1.5-G1642X was degraded (0.64 ± 0.11 arbitrary units) compared with the WT. Therefore, this explains why the current reduction was not of 75%. We also measured the persistent sodium current I_Na–late_ since an increased in persistent current is widely observed in HF. However, we found that Na_v_1.5-G1642X does not lead to an increase in the I_Na–late_ (*n* = 7) of the WT (Figure 1D). The C-terminus of Na_v_1.5 and the DIV S4 are known to modulate the channel inactivation (Cormier et al., 2002). Since we have previously shown that a channel with defective biophysical properties can modulate the gating properties of the WT channel (Shinlapawittayatorn et al., 2011b; Clatot et al., 2017), we questioned whether the kinetics of the WT channel would be influenced by the splice variant since it is lacking the DIV S4 and the C-terminus. However, we found that neither the conductance (*n* = 47, ns) (Figure 1C), the steady-state inactivation (*n* = 50, ns) (Figure 2A), nor the recovery from inactivation (*n* = 48, ns) (Figure 2B) of Na_v_1.5-WT were influenced by Na_v_1.5-G1642X.

**FIGURE 3:**
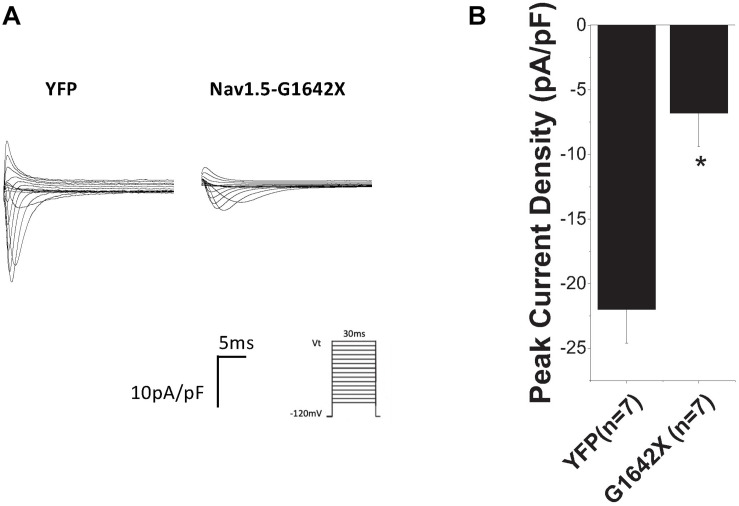
**(A)** Representative sodium current traces. **(B)** Peak current densities recorded from the commercially available iPSC-CM iCells^®^. The control group was cells transfected with YFP and currents recorded from these cells represent the endogenous sodium current present in these cells. This control current was then compared to currents recorded from iCells^®^ transfected with Na_v_1.5-G1642X. Voltage protocol used is included. *p < 0.05 compared to YFP transfected cells.

We then investigated if this DN-effect of the splice variant could be reproduced in human cardiomyocytes, which better mimic what happens in patients. We transfected Na_v_1.5-G1642X into the commercially available iPSC-CMs iCells^®^ and also observed a dramatic reduction (*n* = 7, *p* < 0.05) in endogenous I_Na_ when the cells were transfected with Na_v_1.5-G1642X compared to YFP transfected cells (Figures 3A,B).

### Na_v_1.5-G1642X Is Expressed at the Cell Surface

**FIGURE 4:**
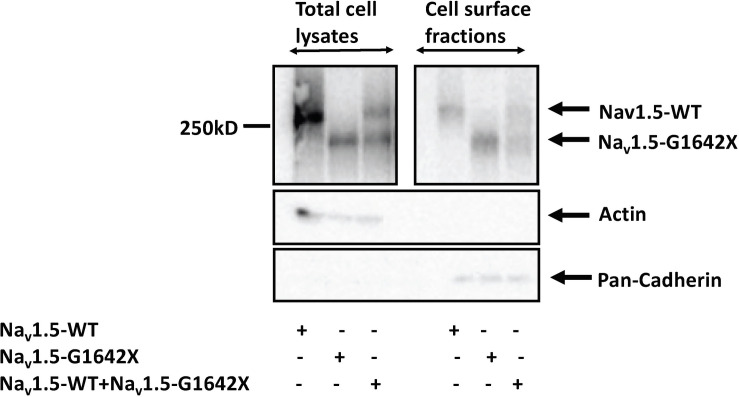
Surface Biotinylation assays showed that the Na_v_1.5-G1642X is expressed at the cell membrane. Na_v_1.5-WT is also present at the cell surface in presence of Na_v_1.5-G1642X. HEK 293 cells were transfected with either Na_v_1.5-WT, Na_v_1.5-G1642X, or Na_v_1.5-WT+Na_v_1.5-G1642X. Western blots were probed with a sodium channel antibody and both bands for Na_v_1.5-WT and Na_v_1.5-G1642X could be distinguished based on the smaller size of the truncated channel.

Other than modulating the channel inactivation, the C-terminus of Na_v_1.5 can bind with multiple partner proteins which can regulate trafficking of the channel (Abriel, 2010). It is thus possible that since this splice variant is missing the whole C-terminus, that this could impair trafficking of Na_v_1.5-G1642X to the cell surface and withhold WT channels inside the cell through their interactions. To explore this possible mechanism of I_Na_ reduction, we measured the expression of the sodium channel at the cell surface through surface biotinylation assays. Similar to the electrophysiological studies, Na_v_1.5-G1642X was co-expressed with the WT to mimic the expression pattern from HF patients. In this experiment, we found that the splice variant Na_v_1.5-G1642X was able to traffic to the cell surface both when expressed alone and when co-expressed with WT. Importantly, the WT was also present at the surface therefore not explaining the DN-effect and reduction in I_Na_ observed (Figure 4). This demonstrates that Na_v_1.5-G1642X doesn’t affect Na_v_1.5 channel trafficking at the cell surface, suggesting that other mechanisms (e.g., biophysical coupling) might be taking place (Clatot et al., 2017, 2018).

### The Coupled-Gating Between Na_v_1.5-G1642X and WT Channels Is Essential to the Dominant Negative Effect

**FIGURE 5:**
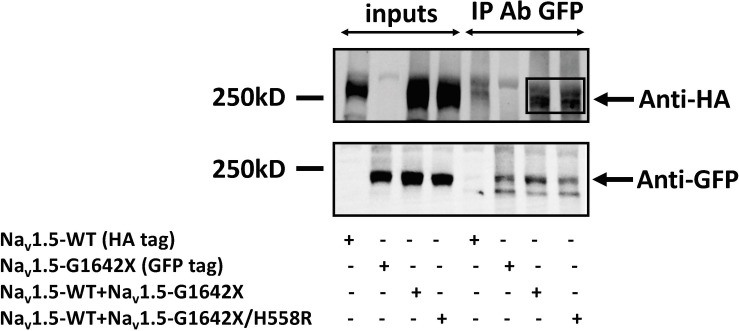
Co-Immunoprecipitation experiments for Na_v_1.5-WT-HA with Na_v_1.5-G1642X-GFP, and Na_v_1.5-WT-HA with Na_v_1.5-WT-G1642X/H558R-GFP. Complexes were coimmunoprecipitated using the anti-GFP antibody and revealed with both anti-GFP and anti-HA antibodies. Bands in the Anti-HA blot for the co-transfection conditions indicate interaction between Na_v_1.5-WT-HA and Na_v_1.5-G1642X-GFP, and between Na_v_1.5-WT-HA and Na_v_1.5-G1642X/H558R-GFP.

**FIGURE 6:**
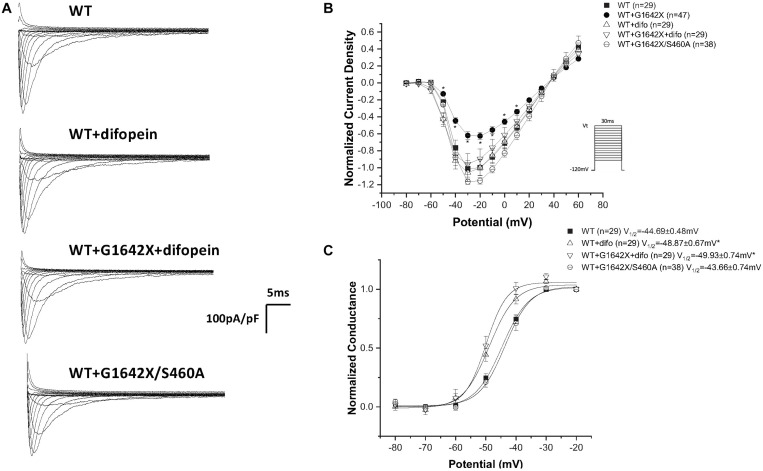
Inhibition of coupled-gating through modulation of 14-3-3. 14-3-3 was inhibited either through difopein (difo) or by mutating serine 460 to alanine (S460A) which removes Na_v_1.5 coupled-gating. **(A)** Representative sodium current traces. **(B)** Current Voltage Relationships (I/V) curves. **(C)** Voltage-dependent conductance curves for Na_v_1.5-WT, Na_v_1.5-G1642X, Na_v_1.5-WT+Na_v_1.5-G1642X, Na_v_1.5-WT+difo, Na_v_1.5-WT+Na_v_1.5-G1642X+difo, Na_v_1.5-WT+Na_v_1.5- G1642X/S460A recorded from HEK 293 cells. Voltage protocol used is included. *p < 0.05 compared to Na_v_1.5-WT. The Na_v_1.5-WT+Na_v_1.5-G1642X I/V curve shown in the right panel is reproduced from Figure 1 for easy comparison.

Our lab previously showed that Na_v_1.5 form dimers through interaction between DI-DII linker (amino acids 493–517), and this dimerization leads to coupled-gating. Similar results for the neuronal voltage-gated sodium channels Na_v_1.1, Na_v_1.2, and Na_v_1.7 were reported by us and other labs (Clatot et al., 2017; Rühlmann et al., 2020). We and others also demonstrated that the protein 14-3-3 is crucial for the regulation of the coupled-gating, and inhibition of 14-3-3 using the inhibitor difopein removed the coupled-gating of the Na_v_s (Clatot et al., 2017, 2018; Rühlmann et al., 2020). In addition, we have shown that the modulation by 14-3-3 is dependent on phosphorylation of Na_v_1.5 Serine 460. Thus, other than direct 14-3-3 inhibition, the 14-3-3 activity and the Na_v_1.5 coupled-gating can be prohibited by mutating this Serine into an Alanine (S460A) (Clatot et al., 2017; Rühlmann et al., 2020). If Na_v_1.5-G1642X leads to a DN-effect through coupled-gating with the WT, we should be able to see the removal of the DN-effect by either using the 14-3-3 inhibitor difopein or with S460A. We first confirmed the interaction between Na_v_1.5-G1642X and the WT with co-IP experiments, and found that Na_v_1.5-WT (tagged with HA) was successfully pulled down by GFP antibody that targets the GFP-fused Na_v_1.5-G1642X (Figure 5). Next, we measured I_Na_ while inhibiting the biophysical coupling of the WT with co-expressed Na_v_1.5-G1642X by difopein administration (*n* = 29, ns) or by mutating Ser460 to alanine (*n* = 38, ns) in Na_v_1.5-G1642X. Importantly, both difopein and the S460A mutation removed the DN-effect of Na_v_1.5-G1642X, restoring current density to the WT alone level (Figures 6A,B). Although difopein did not change the current density, it shifted the voltage-dependent conductance curve toward a negative direction (*n* = 29, *p* < 0.05) (Figure 6C). These results demonstrate that the coupled-gating between the WT and Na_v_1.5-G1642X is essential to the DN-effect and the I_Na_ reduction.

### Na_v_1.5-G1642X Does Not Exert a Dominant-Negative Effect on Na_v_1.5-H558R

**FIGURE 7:**
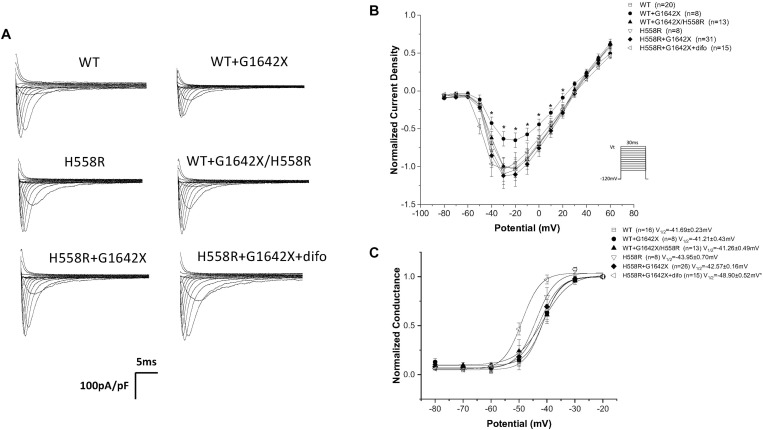
**(A)** Representative sodium current traces. **(B)** Current voltage relationships (I/V) curves. **(C)** Voltage-dependent conductance curves for Na_v_1.5-WT, Na_v_1.5-WT+Na_v_1.5-G1642X, Na_v_1.5-WT+Na_v_1.5-G1642X/H558R, Na_v_1.5-H558R, or Na_v_1.5-H558R+Na_v_1.5-G1642X, and Na_v_1.5-H558R+Na_v_1.5-G1642X+difopein recorded from HEK 293 cells. Voltage protocol used is included. *p < 0.05 compared to Na_v_1.5-WT. The splice variant does not exert DN effect on Na_v_1.5-H558R.

**FIGURE 8:**
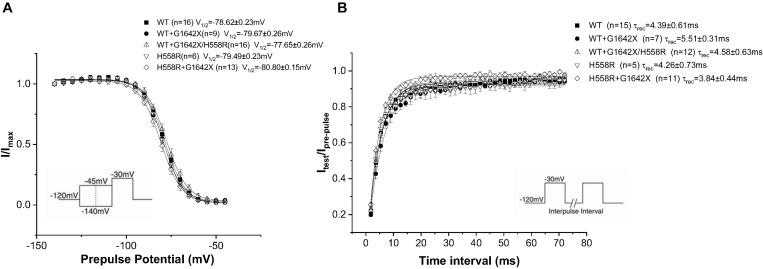
Recovery from inactivation **(A)** and steady-state inactivation **(B)** recorded from HEK 293 cells expressing either Na_v_1.5-WT, Na_v_1.5-WT+Na_v_1.5-G1642X, Na_v_1.5-WT+Na_v_1.5-G1642X/H558R, Na_v_1.5-H558R, or Na_v_1.5-H558R+Na_v_1.5-G1642X. The splice variant Na_v_1.5-G1642X does not influence the recovery from inactivation and the steady-state inactivation of the Na_v_1.5-H558R. Voltage protocols used are included.

Na_v_1.5-H558R is a common polymorphism present in 30% of the population. It is the most frequent Na_v_1.5 polymorphism. This polymorphism is long known to be a genetic modifier of cardiac pathologies. Indeed, H558R can promote the functionality of mutated channels found in BrS, Long QT Syndrome and was also clinically observed to improve the survival of HF patients (Shinlapawittayatorn et al., 2011b; Aleong et al., 2005; Poelzing et al., 2006; Viswanathan et al., 2003; Marangoni et al., 2011; Matsumura et al., 2017). We therefore asked if the Na_v_1.5-H558R polymorphism could improve the survival of HF patients by also impairing the DN-effect caused by the HF splice variant Na_v_1.5-G1642X. We recorded I_Na_ for Na_v_1.5-H558R (*n* = 8, ns), and first tested the effect of Na_v_1.5-G1642X on Na_v_1.5-H558R by co-expressing the two channels together. We found that Na_v_1.5-G1642X did not produce a DN-effect on Na_v_1.5-H558R (*n* = 31, ns) (Figures 7A,C). We also inserted the H558R polymorphism on the G1642X construct (Na_v_1.5-G1642X/H558R) and co-expressed this construct with the WT Na_v_1.5. Interestingly, we found that Na_v_1.5-G1642X/H558R did not produce a DN-effect (*n* = 13, ns) (Figures 7A,B). This suggests that the presence of H558R disrupts the coupling. To confirm this, we also co-expressed difopein which we have shown can disrupt the coupling, with Na_v_1.5-G1642X and Na_v_1.5-H558R, and we did not observe any additional increase in the current density (*n* = 15, ns) (Figures 7A,B). This suggest that the coupling between the Na_v_1.5-H558R and the splice variant Na_v_1.5-G1642X is already disrupted. Difopein as in Figure 6C, also shifted the voltage-dependent conductance curve toward negative voltages (*n* = 15, *p* < 0.05) (Figure 7C). These results therefore suggest that the Na_v_1.5-H558R polymorphism removes this DN-effect by impairing the biophysical coupling between the splice variant and the WT resulting in restored I_Na_. Finally, as observed with the WT, the splice variant did not change the steady-state inactivation (*n* = 13, ns) (Figure 8A) and the recovery from inactivation (*n* = 11, ns) (Figure 8B) of Na_v_1.5-H558R.

## Discussion

Heart failure describes the state of the heart that fails to pump blood. It can cause over 250,000 annual deaths in the United States alone, and the number keeps growing with the aging population. Importantly more than half of patients with HF present with arrhythmias and SCD due to ion channel remodeling (Tomaselli and Zipes, 2004). Electrophysiological remodeling of Na_v_1.5 often occurs in HF resulting in reduction of the peak I_Na_ and an increase in the sustained late sodium current (I_Na__–__late_). Reduction in I_Na_ is also commonly known to associate with BrS and conduction system diseases which can lead to HF and augment the risk of SCD (Valdivia et al., 2005; Remme and Bezzina, 2010; Mizusawa and Wilde, 2012). Splice variants in *SCN5A* naturally occur as a post-transcriptional regulation of Na_v_1.5 expression. To date, Na_v_1.5 splice variants have been found in the sequence of exon 6, exon 17, exon 18, exon 24 and exon 28. These splice variants can be either functional or nonfunctional and they can be developmentally regulated, i.e., exon 6 splice variant is also designated as the neonatal splice variant (Schroeter et al., 2010). The nonfunctional splice variant E28D associated with HF and other cardiac pathologies was shown to contribute to the reduction of INa (Gao et al., 2013; Noyes et al., 2017; Banerjee et al., 2020). In the present study, we found that although associated with HF, the *SCN5A* splice variant E28D (i.e., Na_v_1.5-G1642X) does not contribute to the increased I_Na–late_ observed in HF patients (Figures 1D,E). We therefore explored how Na_v_1.5-G1642X contributes to the reduction of I_Na_ seen in the failing heart. We demonstrated that the truncated channel Na_v_1.5-G1642X interacts with the WT Na_v_1.5 channel and exerts a dominant-negative effect, contributing to the decrease in I_Na_ seen in HF.

The mechanism behind I_Na_ reduction can vary depending on the genetic environment and the pathological condition. Previous studies reported that the reduction in cardiac sodium current can be induced by trafficking deficiency of the *SCN5A* mutants. For example, the mutant *SCN5A*-R282H identified in BrS patients was deficient in trafficking to the cell surface and therefore reduced the cardiac sodium currents (Poelzing et al., 2006). The trafficking efficiency can also be regulated by Na_v_1.5 partner proteins such as ankyrin-G and dystrophin therefore introducing other pathways to disturb the trafficking or surface expression of Na_v_1.5 (Abriel, 2010; Lang et al., 2018). In addition, abnormal post-translational modifications, such as phosphorylation and deglycosylation, were both shown to contribute to the decreased I_Na_ amplitude (Qu et al., 1996; Ufret-Vincenty et al., 2001). Moreover, our group reported that coupled-gating is one of the mechanisms leading to a decrease in I_Na_. We found that the *SCN5A*-L325R BrS mutation exerted a DN-effect through coupled-gating with the *SCN5A*-WT. We revealed that the coupled-gating between the mutants and the WT could reduce the open probability and the coupling of the WT (Clatot et al., 2018). The decrease in sodium current observed in HF and the presence of HF-associated Na_v_1.5 splice variants agree with our previous studies with BrS mutants. Indeed, we show here that the splice variant Na_v_1.5-G1642X interacts with the WT (Figure 5), but has no influence on the channel trafficking, as both the truncated splice variant and the WT are trafficking at the cell surface (Figure 4). It demonstrated that much like BrS mutants, this truncated non-functional channel might exert a DN-effect through coupled-gating with the WT (Figures 1, 3), which would explain the significant reduction in sodium current seen in HF myocytes. When considering a mutant resulting in a DN effect for a dimeric channel, one would expect a reduction in sodium current of about 75% (Clatot et al., 2017). However, unlike what we observed with the BrS DN mutation L325R which leads to a ∼75% I_Na_ reduction (Clatot et al., 2017), the truncated Na_v_1.5-G1642X in the present study reduced the currents by about 40%. One potential explanation would be if there was a mild degradation of the truncated Na_v_1.5-G1642X channel, which is common for truncated proteins. We quantified the expression level of Na_v_1.5-WT and Na_v_1.5-G1642X, and found that about 40% of the truncated Na_v_1.5-G1642X was degraded compared with the WT (0.64 ± 0.11 arbitrary units). Therefore, this explains why the DN effect we observed here is not a 75% reduction in the current density. Nevertheless, consistent with our observations with BrS mutations, we demonstrated that the reduction in current observed here was also due to coupled gating between the two channels. In fact, inhibition of the coupled-gating by modulating 14-3-3 restored the reduced I_Na_ to its WT level (Figure 6).

Our group previously found that the α-subunits of Na_v_1.5 assemble and gate as dimers, and the protein 14-3-3 can regulate the coupled-gating of the Na_v_1.5 (Clatot et al., 2012, 2017, 2018). In our previous study, we also showed that the dimerization site for Na_v_1.5 is located on the DI-DII linker, which is known to be the ‘hotspot’ for Na_v_1.5 phosphorylation (Marionneau and Abriel, 2015). Since the 14-3-3 activity on its target proteins depends on a phosphoserine in a given putative binding motif (Dougherty and Morrison, 2004; Aitken, 2006; Allouis et al., 2006; Lau et al., 2007), mutating S460A on Na_v_1.5 can then modulate the activity between Na_v_1.5 and 14-3-3. Thus 14-3-3 inhibition through difopein and the S460A mutation could both remove Na_v_1.5 coupling. Our data suggest that although inhibiting 14-3-3 with difopein does not produce an obvious effect on the whole-cell current density, it shifts the voltage-dependent conductance curves toward negative voltages. We suspect that the shift is due to the regulation of 14-3-3 on Na_v_1.5 other than the coupling since we do not observe similar shifts through uncoupling with mutation S460A (Figure 6) or H558R (Figure 7). Additionally, we noticed that co-expression of the splice variant Na_v_1.5-G1642X/S460A did not produce a DN-effect on the WT (Figure 6). Importantly, we confirmed that Na_v_1.5-G1642X/S460A is also a non-functional channel (data not shown), which means that the S460A mutation does not lead to a gain-of-function effect which would explain the recovery of the functionality of Na_v_1.5-G1642X. It therefore appears that phosphorylation of S460 plays a critical role in the coupled-gating of Na_v_1.5 and could become an important target to inhibit DN-effects caused by BrS mutations or defective splice variants found in HF. The Na_v_1.5 serine 460 has been identified as a phosphorylated site by mass spectrometric experiments (Marionneau et al., 2012; Iqbal and Lemmens-Gruber, 2019). Therefore we speculate that phosphorylation of S460 is important for biophysical coupling and/or dimerization. Further investigations to study Serine 460, its phosphorylation and its role in the Na_v_1.5 coupling are currently underway in our lab. Our preliminary data do suggest an important role of the PKA phosphorylation in the Na_v_1.5 coupling since the PKA inhibitor KT5720 removed the DN effect caused by DN mutations (data not shown).

The single-nucleotide polymorphism H558R is the most common Na_v_1.5 polymorphism present in the population and has been demonstrated by multiple groups to modify the biophysical properties as well as the trafficking of pathogenic *SCN5A* mutations (Ye et al., 2003; Shinlapawittayatorn et al., 2011a; Makielski et al., 2003; Tan et al., 2005; Poelzing et al., 2006). One of our previous study even showed that using a fragment of only 20 amino acids of Na_v_1.5 containing H558R was able to rescue the trafficking of certain Na_v_1.5 mutants, indicating its definitive capacity in regulating Na_v_1.5 functioning (Shinlapawittayatorn et al., 2011b). Here, the H558R polymorphism was capable of rescuing the Na_v_1.5 loss-of-function resulting from the splice variant DN-effect. Considering that we are seeing similar restoration of currents in presence of H558R as we have seen with 14-3-3 inhibition, and considering the location of the H558R polymorphism is near the dimerization and 14-3-3 binding site, we speculate that a similar mechanism is involved. However, it is beyond the scope of this current study to elucidate this mechanism and future investigations will be focused on this rescuing effect.

Splice variants resulting in truncated channels could easily result in a non-produced or degraded protein which in itself would result in a reduction in current. Indeed, it has also been suggested that these HF-associated *SCN5A* splice variants could reduce the mRNA level of the WT *SCN5A* (Shang et al., 2007). However, the surface expression of the Na_v_1.5-G1642X (Figure 4) that we observed in the current study indicates that the truncated channel can be synthesized by the HEK 293 cells which is also consistent with the results in iPSC-CMs demonstrated by the Dudley group (Shang et al., 2008; Gao et al., 2013). This therefore supports that the mechanism we unveiled here showing a DN-effect between the splice variant and the WT due to biophysical coupling contributes, at least in part, to the I_Na_ reduction observed in HF patients. It is important to note that the ability to reproduce this DN-effect in human iPSC-CMs further support this mechanism in HF patients’ myocytes and explain the reduction in sodium current. Interestingly, we noticed that the reduction in I_Na_ in these cells (Figure 3) is even more significant than that of HEK 293 cells (Figure 1). One can speculate that in human iPSC-CMs, other splice variants such as the neonatal splice variant Na_v_1.5e may be more predominant due to the immature nature of the iPSC-CMs and might be more sensitive to this interaction. It is also possible that contrarily to HEK 293 cells where we control the level of expression of both the WT and the splice variant through the co-transfection of the same quantity of DNA, the ratio of the splice variant to the endogenous channel could be greater which would explain the larger reduction in current.

The Dudley group first demonstrated the upregulation of the splice variant E28D in heart failure which resulted in a decrease in sodium current (Shang et al., 2007). Here we demonstrated that this splice variant produces a DN-effect on the WT sodium channel through interaction with the channel and coupled-gating, further explaining how the splice variant can lead to a reduction in I_Na_ in HF patients. Future studies focusing on developing strategies to uncouple Na_v_1.5 without disturbing I_Na_ might be beneficial to all patients suffering from phenotypes related to *SCN5A* mutants or splice variants.

## Data Availability Statement

The raw data supporting the conclusions of this article will be made available by the authors, without undue reservation.

## Author Contributions

YZ performed the experiments, collected the data, performed the analysis, and wrote the manuscript. XW, DY, and HL performed the experiments, collected the data, performed the analysis, and reviewed the manuscript. AR-N collected the data and reviewed the manuscript. J-DF conceived the study and reviewed the manuscript. ID conceived the study, and wrote and reviewed the manuscript. All authors contributed to the article and approved the submitted version.

## Conflict of Interest

The authors declare that the research was conducted in the absence of any commercial or financial relationships that could be construed as a potential conflict of interest.
